# Orchestration versus bookkeeping: How stakeholder pressures drive a healthcare purchaser’s institutional logics

**DOI:** 10.1371/journal.pone.0258337

**Published:** 2021-10-13

**Authors:** Bart A. C. Noort, Taco van der Vaart, Kees Ahaus

**Affiliations:** 1 Faculty of Economics and Business, Department of Operations, University of Groningen, Groningen, The Netherlands; 2 Health Services Management and Organisation, School of Health Policy & Management, Erasmus University Rotterdam, Rotterdam, The Netherlands; Waikato Institute of Technology, NEW ZEALAND

## Abstract

**Background:**

Healthcare purchasers such as health insurers and governmental bodies are expected to strategically manage chronic care chains. In doing so, purchasers can contribute to the goal of improving task division and collaboration between chronic care providers as has been recommended by numerous studies. However, healthcare purchasing research indicates that, in most countries, purchasers still struggle to fulfil a proactive, strategic approach. Consequently, a typical pattern occurs in which care improvement initiatives are instigated, but not transformed into regular care. By acknowledging that healthcare purchasers are embedded in a care chain of stakeholders who have different, sometimes conflicting, interests and, by taking an institutional logics lens, we seek to explain why achieving strategic purchasing and sustainable improvement is so elusive.

**Method and findings:**

We present a longitudinal case study in which we follow a health insurer and care providers aiming to improve the care of patients with Chronic Obstructive Pulmonary Disease (COPD) in a region of the Netherlands. Taking a theoretical lens of institutional logics, our aim was to answer ‘how stakeholder pressures influence a purchaser’s use of institutional logics when pursuing the right care at the right place’. The insurer by default predominantly expressed a bookkeeper’s logic, reflecting a focus on controlling short-term care costs by managing individual providers. Over time, a contrasting orchestrator’s logic emerged in an attempt to achieve chain-wide improvement, striving for better health outcomes and lower long-term costs. We established five types of stakeholder pressure to explain the shift in logic adoption: relationship pressures, cost pressures, medical demands, public health demands and uncertainty. Linking the changes in logic over time with stakeholder pressures showed that, firstly, the different pressures interact in influencing the purchaser. Secondly, we saw that the lack of intra-organisational alignment affects how the purchaser deals with the different stakeholder pressures.

**Conclusions:**

By highlighting the purchaser’s difficult position in the care chain and the consequences of their own internal responses, we now better understand why the intended orchestrator’s logic and thereby a strategic approach to purchasing chronic care proves unsustainable within the Dutch healthcare system of managed competition.

## Introduction

Healthcare purchasing organisations (hereafter: purchasers), such as health insurers and governmental bodies, are expected to achieve the right care at the right place through strategic purchasing, which the WHO defines as involving “a continuous search for the best ways to maximise health system performance, by deciding which interventions should be purchased, how, and from whom” [1]. Although healthcare policies, regulation and financial incentives based on the principles of managed competition are designed to drive strategic purchasing, earlier studies have noted that purchasers often struggle to meet this goal [2–5]. Indeed, research and practice often show a typical pattern of improvements and innovations being initiated, but not sustainably translated into regular care [6–8]. Here, opportunities are being missed since there is still much room for improving care delivery and outcomes, particularly for chronically ill patients. This paper therefore questions to what extent and under which circumstances purchasers are able to initiate, stimulate and support sustainable improvement in care delivery, and thus act as a true strategic purchaser. We posit that, despite their efforts to become a strategic purchaser, purchasers are inclined to fall back into a more traditional role as the bookkeeper of their care chains. We anticipate that such patterns can be explained by the demands exerted by various stakeholders such as physicians, patients and care-provider managers. Understanding how the purchaser’s strategies and actions are affected over time by the care-chain context in which they operate will help purchasers and policymakers in their aim of achieving the right care at the right place. Here, we take the theoretical lens of institutional logics to longitudinally study when, how and why stakeholder pressures influence purchasers in this pursuit.

This paper adopts a chain-wide perspective to explain why purchasers struggle to actively manage care providers, which policymakers seem to expect, and what shapes a purchaser’s strategy. Recognising that what drives the actual actions, behaviours and strategies of an organisation is a subtle process involving multiple actors with different beliefs and responsibilities, we go further than most of the healthcare purchasing literature which limits itself to describing financial, regulatory or relational tools [4,9,10]. The theory of institutional logics explains that it is common within organisations for different practices and ways of thinking to emerge related to the various external and internal pressures and interests [11,12]. Such institutional logics are defined in the values, practices, beliefs and assumptions that shape the cognition and decision-making of people and organisations [11]. Research tells us that organisations tend to sometimes develop conflicting institutional logics, such as commercial versus professional logics [12–14]. Also, studies show how institutional logics may change, and that there are different ways in which organisations deal with tensions between logics [15,16]. Here, the lens of institutional logics has proven a valuable perspective in explaining the behaviour of organisations in dealing with the demands of various stakeholders. By using this framework, we are able to improve understanding of how the different policies, interests and involved stakeholders shape the purchaser’s strategy and how this develops over time. Through this, we contribute to the healthcare purchasing literature which has recognised the challenges of adopting managed competition principles, but has so far only paid attention to healthcare purchasers’ actual behaviour in a real-life setting [2–4,17,18].

We were given the opportunity to longitudinally follow a health insurer pursuing better task division and collaboration between providers of Chronic Obstructive Pulmonary Disease (COPD) care. The purchaser, a large health insurer operating in the Dutch healthcare system of managed competition, had the goal of adopting what we call an orchestrator’s logic, as such resembling strategic purchasing as defined by Klasa et al. [4]. This logic contrasts with the purchaser’s bookkeeper’s logic that relates to the traditional responsibility for controlling costs within annual budgets. In this study, we longitudinally follow a purchaser’s adoption of these logics. Through this, we aim to understand how a purchaser´s strategies and actions unfold over time, how these are shaped by stakeholder pressures, and finally the patterns that emerge over time to explain why initiated improvements for chain-wide care delivery are so hard to sustain.

## Scientific background

### Managing chronic care chains

Improving chronic care delivery requires careful allocation of expertise, tasks and responsibilities, supported by referral guidelines and appropriate systems for information exchange [19–22]. Further, it is essential to structure the collaboration between providers through for example, regular inter-professional consultations and shared treatment plans [20,21,23]. However, achieving such improvements is difficult due to conflicting financial interests and the different medical capabilities and viewpoints of providers. By managing contracts and relationships with providers, the purchaser can play an important role in overcoming these difficulties.

The current ways of paying providers for services do not incentivise providers to pursue better task division or collaboration [24]. This is because the various providers are contracted separately, and payment is usually on a fee-for-service basis. Such payment systems do not incentivise shifting tasks to other providers or reducing avoidable care interventions. To resolve this problem, several studies have proposed contractual forms that incentivise improvements such as pay-for-performance [25], shared savings [26] and bundled payments [24]. Nevertheless, designing such innovative contracts has proven difficult and they may have unintended effects [27]. Steering through financial incentives, such as long-term agreements on new payment schemes, remains hard to achieve [28].

Besides contracts, purchasers have other means through which they can manage their care chain. A common steering mechanism is to monitor the provider’s performance using quality and cost indicators [29]. This transparency-based mechanism is particularly effective if patients have freedom to choose providers as the purchaser can try to direct patients towards the best-performing providers [9,30]. Another way to influence care provision is what Sheaff et al. [29] call ‘micro-commissioning’, where purchasing employees become closely involved in discussing how providers should organise and deliver their services. This mechanism relies heavily on a close relationship between the purchaser and provider(s) and the willingness of the parties to collaborate [29]. Other studies show that, through building a trustworthy long-term relationship with providers, purchasers can achieve care chain improvement [31–34]. However, building such relationships is challenging, and conflicts and lack of trust are often reported as a consequence of goal and power struggles between the parties [5,32]. Although there are examples where purchasers have structurally provided the appropriate financial incentives and invested time and effort in achieving care chain improvement [35,36], these tend to be incidental, temporary or on a limited scale. Generally, purchasers appear to have an administrative, short-term approach and contract individual providers rather than provider networks [5,32].

Currently, the expectation is that individual health insurers will use purchasing mechanisms to achieve better health outcomes and thereby lower costs in the long run, leading to lower insurance premiums and possibly a larger market share. Nevertheless, one has to recognise that purchasers have to deal with multiple stakeholders including patients, physicians, managers, and governmental bodies. One stakeholder’s interests can conflict with those of the purchaser, or with those of other stakeholders, placing the purchaser in a difficult position. Healthcare management and services scholars have recognised this difficulty, showing the elusiveness of realising strategic purchasing [2–5,17,18]. Given the purchaser’s difficult position, one could argue that, based on Transaction Cost Economics (TCE), it is indeed challenging and possibly unrealistic to invest time and take financial risks to improve long-term population health gains [18,37]. At the same time, we believe there is still much to be learnt on how purchasers could develop their way of managing chronic care chains, particularly in terms of the circumstances and timing of their actions. Studying when, why and how the different stakeholders exert pressure on the purchaser will help to understand a purchaser’s strategies and actions, and the longitudinal setting of this study can provide an understanding of how this develops over time.

### Institutional logics in organisations

Organisations adopt institutional logics that are shaped by the goals, visions and interests of actors within the organisation, but also through pressures from external stakeholders [11–13,38]. Multiple institutional logics can be present within an organisation and studying these logics can help to explain the organisation’s behaviour. Several mechanisms have been identified that organisations use to deal with different, sometimes conflicting, institutional logics. Commonly, when a new institutional logic is introduced in an organisation, a period of conflict occurs, resulting in a dominant logic emerging [39]. Mechanisms of compromise are also recognised, where a middle ground between two logics is found [39]. The decoupling mechanism reflects a more symbolic way of externally expressing a new logic while, in practice, the organisation sticks to its old logic [12,39].

While studying how work reintegration evolves in companies, Pache and Santos [16] identified the mechanism of ‘selective coupling’, which appeared to be an intermediate between compromising and decoupling. Here, organisations invoke different elements of conflicting institutional logics and are thereby able to follow both ‘old’ and ‘new’ logics demanded by external stakeholders [16]. Similarly, McPherson and Sauder [15] showed that, within organisations, people will sometimes flexibly use different institutional logics. The so-called ‘hijacking’ mechanism reflects how, depending on the goals being pursued in a particular situation, persons within organisations appear willing to sometimes invoke logics which conflict with their own [15]. These two studies show that while individuals and organisations have values and beliefs that normally determine their actions and decision-making, they can, on occasions, pragmatically deal with problems, interests and pressures related to surrounding stakeholders by showing behaviour that does not match their institutional logic. Here, the lens of institutional logics can explain why organisational behaviour of healthcare purchasers may vary in different situations and over time.

Institutional logics, and thereby the behaviour of healthcare purchasers, are shaped by the stakeholder pressures they encounter when managing care chains. Such pressures can occur when purchasers try to collaborate with care providers to set up innovative projects, or while making agreements on task division and collaboration between multiple providers along a care chain. In this study, we expect a focal purchaser to initially express a bookkeeper’s logic, with a lack of strategic purchasing, as shown by Klasa et al. [4]. However, based on the purchaser’s expressed goal of pursuing the right care at the right place in the COPD care chain, the emergence of an orchestrator’s logic was expected. Over time, purchaser’s perceived goals and how to achieve them are likely to be influenced by stakeholder pressures encountered during the process. Applying the institutional logics lens contributes to better understanding the purchaser’s struggle to adopt a strategic approach in managing care providers.

## Methodology

### Research design

We have conducted an in-depth, longitudinal case study in order to gain a thorough understanding of the purchaser’s role in pursuing the right COPD care at the right place. The single case study approach is seen as a suitable strategy for exploration and theory building/elaboration, and is appropriate since there is only limited knowledge about the purchaser’s role in managing healthcare chains [40]. We have chosen a longitudinal approach as we want to understand how the purchaser’s adoption of institutional logics changes over time, and how stakeholder pressures influence this logic adoption. Moreover, a longitudinal, observational case study design avoids the recall bias that may occur in retrospective studies. We conducted the research in a setting where the purchaser had expressed an intention to be actively involved in improving COPD care delivery, with a particular focus on improving task division and collaboration between primary and secondary care providers. Our intention was to gain a thorough insight into the role of the purchaser by collecting data from multiple involved subjects at different points in time. Perceptions and actions were extracted from the data, from which we were able to identify the conflicting bookkeeper’s and orchestrator’s institutional logics invoked by the health insurer. With respect to stakeholder pressures, we identified the interests and goals expressed and pursued by the various stakeholders. We aimed to understand how the purchaser’s involvement affected efforts aimed at improving task division and collaboration between the different care providers.

### Research setting

The research setting is the COPD care chain in a Dutch region with 600,000 inhabitants. The main actors in this study are a pulmonology partnership operating in four hospitals, about 300 general practitioners (GPs) and the leading health insurer to which 70–80% of the region’s inhabitants subscribe. Physiotherapists, community and mental care providers are also involved in caring for COPD patients. The care chain can be characterised by a strong division between primary and secondary care. There have already been several attempts to improve task division and service quality, but joint initiatives have been rare or unsuccessful. Such attempts include developing standardised protocols, individualised treatment plans and follow-up care after hospitalisation.

The Dutch healthcare system is based on the principles of managed competition, where independent care providers are generally paid on a fee-for-service basis by private health insurers, with national governmental bodies acting as regulators [3,41]. These healthcare system characteristics, along with the challenge of improving care in a complex chronic disease chain, provide a typical case in which to study a private purchaser’s attempts to initiate, stimulate and support care improvements through adopting a strategic purchasing approach.

Prior to the start of this study (in 2015), the insurer had started to develop a strategy aimed at actively managing and improving care provision in the region. The goal of this purchaser was to improve care quality and care outcomes, and thereby reduce long-term costs. Part of this strategy is to give GPs more responsibility for providing care to chronically ill patients and improving collaboration between primary and secondary care providers. For COPD, the purchaser-initiated meetings with providers to discuss the organisation of the care chain. These meetings addressed the roles and responsibilities of the different care providers, ways to improve communication and collaboration and financial implications. At the beginning of 2015, these initiatives evolved into a COPD out-of-hospital coaching project which was set up in collaboration with the pulmonology partnership. This project was aimed at improving follow-up care after hospitalisation for patients with severe COPD. Providing out-of-hospital coaching by hospital nurses was expected to improve the connection between primary and secondary care and reduce re-hospitalisations through better recognising patient needs and giving training in self-management skills, as previously reported in similar interventions [42,43]. A project team consisting of the insurer’s managers, pulmonologists and hospital managers was established to discuss and plan this initiative. In addition to this project, other regular meetings between the insurer’s managers, pulmonologists and GPs continued in order to discuss ongoing efforts to improve the COPD care chain. The authors of this paper participated as observers in the project team meetings and were also frequently invited to other meetings.

An important aspect of the project was recognising that the current care reimbursement system does not provide financial incentives for improving COPD care delivery and outcomes. First, providers are paid on a fee-for-service basis, which lacks an incentive to reduce preventable care or to shift care to other providers when appropriate. Second, each care provider is paid separately, which encourages care professionals to focus on delivering services themselves and less on collaborative care arrangements. The purchaser and the providers all recognised this problem, and therefore it was agreed to support the project with financial incentives through shared savings contracts.

### Consent and ethical approval

The COPD out-of-hospital coaching project received medical-ethical review and approval, conducted by the Regional Assessment Committee for Patient-related Research. As part of this review, the collection of qualitative data as reported in this paper was also approved by the committee. Eligible patients (hospitalised adults with severe COPD) for the out-of-hospital coaching project were informed about the study by one of the coaches, received verbal and written information, were asked to sign a consent form and had a more elaborate introductory meeting during hospitalisation. All interviewees gave written consent to participate in this study. The consent form provided information about the study and handling of the data and was documented by the first author of this paper.

### Data collection

The unit of analysis is the regional COPD care chain. Starting in April 2015, we collected observation data by attending meetings related to the improvement initiatives in the studied region (19 meetings to date, see Table 1). These meetings were attended by the purchaser’s employees, medical professionals, hospital managers and all the authors of this paper. The main aims of the meetings were to discuss possible initiatives to improve COPD care, the implementation of the out-of-hospital coaching intervention, and to discuss financial and other agreements. The authors of this paper were involved in the scientific evaluation of the COPD out-of-hospital coaching intervention and observed and studied, without influencing, the collaborative process between attendees. Field notes were made about the negotiation process, decisions made, problems encountered and the goals and interests of the different parties.

**Table 1:** Project team meetings and related meetings.

Meeting	Date	Number of attendees
1. Project team meeting	May 2015	6
2. Project team meeting	June 2015	9
3. Project team meeting	June 2015	8
4. Regional meeting with care providers	July 2015	~30
5. Project team meeting	August 2015	8
6. Project sub-team meeting	August 2015	5
7. Project team meeting	September 2015	10
8. Pulmonology partnership meeting	January 2016	~20
9. Project team meeting	March 2016	11
10. Project team meeting	June 2016	10
11. Intervision meeting with coaches	October 2016	12
12. Project team meeting	October 2016	9
13. Intervision meeting with coaches	February 2017	12
14. Project team meeting	March 2017	9
15. Project team meeting	November 2017	11
16. Project team meeting	July 2018	11
17. Intervision meeting with coaches	July 2018	12
18. Project team meeting	Oct 2019	9
19. Intervision meeting with coaches	Dec 2019	11

Attendees–health insurer: Contracting managers, policy advisors, medical advisors, financial controller; care provider: Board member, department manager, medical specialist (pulmonologist), general practitioner, nurse, physiotherapist; patients: A member of a patient representative organisation; plus one independent project manager.

Between March and May 2016, the first author of this paper held 12 semi-structured interviews with people involved in the improvement projects (Table 2). During the interviews, we reflected on the project meetings and key events to gain an understanding of the goals, interests, problems and decisions. Also, we asked interviewees to explain the main issues related to improving task division and collaboration in the care chain. We asked interviewees to reflect on how and why the purchaser expressed certain actions, perceptions, beliefs and assumptions (see S1 File for the interview guide). Through these questions, we gained a thorough understanding of both the stakeholder pressures and the purchaser’s institutional logics. By observing and interviewing subjects on both the purchaser and the provider sides, we obtained a balanced view on care delivery issues and the purchaser’s role as manager of the care chain. Between March and November 2017, a second round of 13 interviews was conducted to update our insights into the progress of the projects, and agreements and actions made by the purchaser. Data triangulation was conducted by analysing secondary data from management reports, care protocols, presentations and reports on regional demographics.

**Table 2:** Interviews related to the COPD improvement initiatives (interview duration 30–90 minutes).

Organisation	Function	Number of interviewees round 1	Number of interviewees round 2
Hospital	Board member	2	
	Department manager	2	
	Pulmonologist	2	1
	Trauma physician		1
Health insurer	Policy advisor	2	1
	Contracting manager	1	2
	Financial controller		1
	Medical advisor		1
Physiotherapist	Physiotherapist	1	1
General Practitioner	General Practitioner	2	2
	General Practice nurse		1
Other	Emergency Department manager		1
	Project manager		1
Total		12	13

### Analysis

Our research strategy is based on the Gioia methodology [44] which starts with inductive data analysis, but also uses deductive (or ‘abductive’) reasoning by drawing on the literature. We opted for this strategy as we were uncertain about the institutional logics that we were seeking, which stakeholders would be involved and what institutional pressures they would exert. Coding was performed by the first author of this paper. Initial and final findings, as well as when data saturation was achieved, were iteratively discussed between all authors. The collected data were analysed by first sticking as closely as possible to the informant’s terms, thereby creating a long list of first-order codes (using ATLAS.ti and MS Excel). These were placed in two first-order categories: 1) the purchaser’s institutional logics, based on their actions, perceptions, beliefs and assumptions, and 2) stakeholder pressures on the purchaser based on the stakeholders’ actions and interests. After the first coding process, we constructed a case narrative to gain a better understanding of the data and to identify initial patterns to explain how stakeholder pressures influence the purchaser’s institutional logics. During the second-order coding, we aggregated the first-order codes. Establishing the logics employed involved a detailed discussion among the authors based on constructed descriptions of ‘ideal types’ of logics [45]. In this way, we could refine the initial candidate logics. As the third and final step, we sought patterns in the data by studying how the stakeholder pressures were related to the purchaser’s adoption of each of the institutional logics. Findings were triangulated by means of secondary data such as policy documents and regional care guidelines. All the interviews were coded in Dutch, relevant quotes and passages were then translated into English.

## Findings

### Defining the orchestrator’s and bookkeeper’s logic

As a starting point, we aimed to establish and define the competing orchestrator’s and bookkeeper’s logics within the purchaser. Through an iterative process, we gained a thorough understanding of both logics that assisted us in studying and understanding the purchaser’s behaviour. We further defined and confirmed the logics by using the ideal-type framework shown in Table 3 [45]. In essence, the bookkeeper’s logic is characterised as a cost-focused, short-term and single-provider approach to contracting healthcare services. In contrast, the orchestrator’s logic reflects a health-focused, long-term and chain-wide strategy. In the next section, we first provide an overview of the setting up of the coaching project and ways to achieve the right care at the right place. We discuss the purchaser’s actions, perceptions, beliefs and assumptions, from the perspective of the multiple parties involved. We then present explanatory mechanisms that provide an understanding of how stakeholder pressures influence the adoption of either the bookkeeper’s or the orchestrator’s logics.

**Table 3:** Ideal types of the identified bookkeeper’s and orchestrator’s institutional logics.

Characteristic	Bookkeeper	Orchestrator
**Sources of organisational legitimacy**	Health insurer as controller of public care budget	Health insurer as a driver of quality and effective care for patients
**Target of legitimacy pursuit**	The health system, regulation	Public and local community interest
**Basis of organisational mission**	Self-focussed: Enforcing providers to stay within budget Demanding providers to conform to regulation Build a competitive position Increase profits and cash flow	Supply chain wide: Flexibility in budgets Joint problem solving Managing, supporting providers Driving collaboration between providers
**Basis of organisational attention**	Focus on the firm’s market position Compliance and adherence to regulations	Focus on public interest Patient perspective
**Basis of strategy**	Annual contracting cycle	Ongoing efforts to solve problems, establish innovation/change projects
**Primarily associated actors**	Purchaser’s general managers and contracting managers	Policy managers Medical advisors Strategy advisors

### The emergence of the orchestrator’s logic and falling back into bookkeeping

We were able to distinguish three phases during the period studied: (1) the emergence and (2) subsequent dominance of the orchestrator’s logic within the purchaser, and finally (3) falling back into the default bookkeeping logic.

### Phase I: From bookkeeping to orchestration (pre-2014)

The bookkeeping logic within the insurer reflected the contracting cycles between the purchaser and care providers. At that time, most contracts between insurers and providers in the Netherlands had a one-year term and negotiations were focused on overall costs and volumes of services. Conflicts frequently occurred with care providers claiming that their care budgets were insufficient, sometimes leading to stopping patient treatment. Provider managers and doctors complained that contract negotiations should be based more on medical content and quality of care, and less on costs.

*“I have the feeling that it usually goes like this*: *the health insurer says*, *OK this was the budget last year*, *this will be reduced by 10%*. *Well*, *there is little medical input to that*.*”* Pulmonologist 1

This purchaser’s bookkeeper’s logic can be explained by the need to remain financially solvent to be able to deal with potential future cost increases. Also, increasing outgoings in the short-term requires a corresponding increase in insurance premiums, which could reduce the client base. Furthermore, negotiations based on medical content and quality are time-consuming since hospital offerings are broken down into 5,600 different service products (Diagnosis-Related Groups, DRGs).

The purchaser seemed to be becoming increasingly aware of the limitations of the short-term contracting cycles. Further, to support their intended strategy of shifting some tasks from hospitals towards primary care, the CEO of the purchaser announced the aim of signing shared savings type of contracts, meaning that care providers could benefit whenever they realise cost reductions.

*“As part of these contracts*, *we look at substitution*, *which care services can go where*?… *We are on the eve of pushing changes*. *It is no longer a question of if we want to do that*. *It needs to be done…As part of our Vanguard status*, *contract innovation is possible*, *for example we work with shared savings agreements in mental care and surgical care”* Interview in news media with CEO of Health Insurer 2015

Such expressions by the purchaser were considered significant by the involved physicians and were drivers for starting meetings on improvement initiatives. The recognition of the advantages of the orchestrator’s logic can be explained by the purchaser’s awareness that providers needed to be stimulated to improve by offering them financial incentives and reducing their financial uncertainty. Also, long-term contracts could reduce transaction costs by removing regular repetitive negotiations.

The emergence of the orchestrator’s logic is also reflected in the purchaser’s initiation of a regional innovation programme aimed at subsidising improvement initiatives. Through this programme, the purchaser set up a committee of doctors, provider managers and purchaser employees to jointly review initiatives aimed at care chain improvement. For the purchaser, the programme provided a way to improve care that could lead to long-term reductions in care costs. The programme, by showing the purchaser’s willingness to fulfil an orchestrator’s role, was also aimed at improving their public image that might attract new policyholders.

### Phase II: Orchestrating the COPD chain (2014 –mid-2015)

The orchestrator’s logic is expressed in the COPD out-of-hospital coaching project that was set up in collaboration with the pulmonology partnership (operating in four hospitals). The aim was to prevent COPD-related re-hospitalisations through supporting patients in coping with their disease and strengthening the provider network. A project team was organised involving the purchaser’s employees, pulmonologists and hospital managers. During joint meetings, it was agreed to initiate a ‘COPD coaching’ project in which hospital-based pulmonology nurses would conduct a series of home visits after hospital discharge.

The reason the purchaser was interested in this project was the potential to prevent further hospitalisations and thereby reduce costs and improve health and patient satisfaction. In addition, such a project as part of the regional programme, if successful, could boost the purchaser’s public image. During the planning stage of the project, the purchaser’s employees involved showed considerable commitment by taking part in discussions on the medical content of the project and by supporting the development of a project plan. The purchaser also demonstrated the orchestrator’s logic by addressing the pulmonologists’ concerns about possible negative financial consequences for their partnership and hospitals. One of the purchaser’s employees commented that a shared savings contract would be a realistic option for this project.

*“As a hospital*, *you will see a reduction in turnover [with improving care outcomes]…well*, *that is not a positive stimulus…we want to achieve behavioural changes*…*so I would like to offer a shared savings [contract]*.*”* Purchaser Policy Advisor 1

In planning the coaching project, the purchaser showed a chain-wide approach. Initially, the pulmonologists focused on improving patient support based on patient knowledge and the expertise of the hospital nurses. The purchaser’s employees, however, frequently mentioned the importance of involving GPs and physiotherapists in the project. Consequently, additional focus was put on communications between pulmonology nurses and primary care providers. Further, a GP and a physiotherapist representative were added to the project team. Furthermore, the nurses were instructed to motivate patients to start physiotherapy, for which the purchaser also reserved funds in case this was not covered in a patient’s insurance package (even for patients from competing health insurers). This involvement by the purchaser reflects its interest in designing a project based on best medical practice, but also their awareness of taking other stakeholder interests into account in order to ensure the long-term success of the initiative.

*“The success of such a project depends on the commitment of GPs as they will be the ones to send the patient to the pulmonologist*. *I think we should invite GPs and representatives from primary care groups”* Purchaser Policy Advisor 1, during a project team meeting, June 2015

*“From the beginning I thought*: *should this be a project only for the pulmonologists*, *shouldn’t other parties be involved*?*”* Purchaser Policy Advisor 1, first interview

As a result of the frequent interactions and collaboration during the coaching project and meetings concerning care chain improvements, the purchaser–provider relationship improved. This subsequently reinforced the purchaser’s ongoing strategy. For example, during the process of setting up the COPD coaching project, the purchaser first appeared reluctant to invest in the project and demanded a strong business case. However, as the talks between the purchaser’s staff, physicians and hospital managers continued, we observed an increasingly flexible attitude by the purchaser. Even though delivering a strong and positive business case remained a challenge, the purchaser’s employees appeared willing to defend the business case within their organisation in order to warrant continuation of the project. The purchaser’s staff saw the developing and improving relationships with the pulmonologists, GPs and hospital managers as an important reason to invest in the COPD coaching project.

*“As the meetings continued*, *I perceived we came a bit more together…because of the trust created by some of the participants”* Pulmonologist 2

*“I found it good to see that there are medical specialists who dare to look beyond their own interests*. *That*, *I found really nice about this [COPD coaching project]*. *And*, *for me personally*, *that means that I put a bit of extra effort into arranging things”* Purchaser Policy Advisor 2

### Phase III: Falling back on the bookkeeper’s logic (mid-2015–2019)

In the summer of 2015, the project plan and business case had been completed and submitted for approval by the programme committee. This triggered discussions between the purchaser and providers about the required financial contributions from the parties. The providers expected the purchaser to fund the improvement project, especially since it could lead to future cost savings for the purchaser. The purchaser, however, expressed a bookkeeper’s logic by emphasising the financial risks related to the uncertain outcomes of the project and the provider’s own responsibilities for realising better quality care. Tensions related to the COPD coaching project, as well as other initiatives, reached such a high level that, eventually, the provider’s managers stepped out of the regional innovation programme. Consequently, the intended incentive schemes such as a shared savings contract were no longer discussed. In contrast to the purchaser’s initial intentions, we observed disunity as several of the purchaser’s staff had moral reservations about financially incentivising care improvements.

*“Within our organisation*, *there are varied opinions about shared savings*, *so I don’t feel free to offer such a contract*. *We should first bring that discussion to a good end internally*, *and we are not there yet*. *There is a group who says*: *But why then*? *They [care providers] should just do what we agreed with each other”* Purchaser Policy Advisor 1, first interview

*“Usually this is hard*, *because what are shared savings*? *How are you going to spend this*? *Usually*, *you will lose money when you look at the bigger picture”* Purchaser Contracting manager

Despite the managerial conflicts, the purchaser’s management decided to still support the coaching project and met the project budget (€0.5M) without a financial contribution from the providers. Reasons for this decision were as before: the purchaser wanted to show publicly that they could deliver a successful project and they had faith in the pulmonologists’ intentions. Following the investment decision, the project team started to implement the coaching intervention. Although the implementation went well, confusion initially emerged about the financial agreement. For the hospitals, it was unclear whether the purchaser was demanding a financial contribution, and if a shared savings contract was still an option. This was particularly problematic for the pulmonology department’s managers responsible for planning daily nursing capacity. With respect to paying for physiotherapy, there appeared to be a lack of awareness within the purchasing organisation of the agreements made for the coaching project. These financial issues delayed the project and revealed the limited support within the purchasing organisation, thereby reflecting a continuing presence of a bookkeeper’s logic. Closely involved purchaser’s employees reflected on these tensions between the emergent orchestrator’s logic and the ever-present bookkeeper’s logic elsewhere in the organisation:

*“We [two managers] are very critical (towards joint projects with providers)*, *other people within our organisation may say `we need to collaborate*, *nice*, *it works`*. *We are a bit more critical*.*”* Purchaser contracting manager 2

“There are hardliners [within our organisation] who say: ‘they [the pulmonologists] make money, why should we let them make more money?” Purchaser Policy Advisor 1

These expressions show that, internally, there was a lack of trust in care providers. In hindsight, the lack of an internal consensus explains why, during the meetings, the purchaser never developed the intended shared savings contract despite frequent requests from the pulmonologists. Providers often mentioned that the purchaser’s inconsistency towards improvement initiatives had a negative impact on the sustainability of improvements and harmed the purchaser–provider relationship.

*“That stagnation in actually making the savings concrete*, *that frustrated me”* Pulmonologist 1

*“I feel there is a lack of a consistent strategy within the purchaser*, *now nobody feels responsible to start talks about agreements on how to make these improvements structural”* Project Manager

As the project developed, we observed a decline in the active chain-wide care management that was initially intended by the purchaser. Although the purchaser had encouraged the pulmonologists and hospitals to involve GPs in the COPD coaching project, this appeared difficult. From a clinical perspective, pulmonologists and GPs have different viewpoints about who should deliver which COPD services. Moreover, changing the task division between providers would affect their incomes. Consequently, the care providers expected the purchaser to contractually support such changes. For the purchaser, it was difficult to manage the differing opinions and interests of the providers. The purchaser’s policy advisors organised several meetings to create consensus among the providers but they lacked the organisational support to translate this into agreements and contracts. Again, we observed a lack of decisiveness within the purchaser, creating stagnation and frustration for the providers.

*“For me*, *it is a bit ambiguous*. *I think we should stimulate improvements but*, *on the other hand*, *it only works when you have enough support*, *so I think the actual energy should come from the field [providers]”* Purchaser Medical Advisor Primary Care 1

*“We have no idea about their agenda on medical content*, *on finance…on their vision…that makes it extremely confusing for us; where does the insurer want to go*? *They speak nice words*, *but when we come with a proposal they seem to delay things*, *there appears to be a total lack of trust in the competence and responsibility of our care professionals and our organisation”* General Practitioner 4

In addition to the difficulties the purchaser had in managing the various providers involved, we also observed that there was an internal, structural reason for the lack of a chain-wide approach. Within the purchaser, there is an organisational split between primary and secondary care. Contracting managers, policy advisors and medical advisors all have responsibilities towards specific provider groups. This organisational construction encourages a formal contractual way of managing care delivery since each department aims to control its own costs by making agreements with a category of individual providers. In other words, the purchasing structure does not support the intended patient-centred or chain-wide approach which includes a long-term financial perspective. The bookkeeper’s logic thus seems to be structurally embedded within the organisation.

*“The first moment I heard about the coaching project was during a meeting with GPs*, *they had understood that we intended to invest in this…they were not happy with that*…*I think it is too bad that internally we didn’t look at this from a broader perspective”* Purchaser Medical Advisor Primary Care 1

*“Medical advisors*, *they are separated from the contracting department (…) and are mostly responsible for determining if patients have the right to certain types of care (…) This depends on their personality and meddlesomeness*, *and it depends on the contracting managers whether the medical advisors are involved”* Purchaser Policy Advisor 1

As a consequence of the purchaser’s failure to create a consensus among providers and the lack of internal alignment, the policy managers took the deliberate decision to continue the COPD coaching project primarily with the pulmonologists. Although medically and patient-wise this was not considered the best way, the purchaser saw this as a pragmatic approach as ‘*it would only complicate the process’* if too many stakeholders were involved.

*“The proposals of the pulmonologists [to organise the COPD coaching project from within the hospitals] seemed reasonable to me…in the end it is their responsibility and not mine*, *or the insurers*. *(…) We try to keep the process as simple as possible*, *to insure we make progress”* Purchaser Policy Advisor 1, first interview

The GPs particularly felt bypassed in the way the COPD coaching project was setup. This further damaged the already tense relationship between the GPs and the pulmonologists, and also between the GPs and purchaser.

*“When I talked with the GPs they found it strange [that they were not involved]*, *they actually found that the pulmonologists had crossed a line*, *like*: *hold on*, *you are going to do things in primary care…why don’t we know anything about that*, *why were we not involved*?*”* Project Manager

The persisting bookkeeper’s logic within the purchaser’s organisation failed to improve the relationships between the various care providers. Purchasing staff often emphasised the conflicting financial interests and medical viewpoints between care providers and they were not confident that they could overcome these conflicts.

Despite all the tensions and start-up problems, the implementation of the project continued. As of mid-2018, about 150 COPD patients were enrolled in the coaching project. A scientific evaluation shows an improvement or at least stabilisation of various clinical measures such as respiratory distress, self-management awareness, depression and anxiety, and nutritional status (preliminary data available upon request). An analysis of reimbursement data indicates a reduction in the number and cost of hospitalisations compared to the year prior to the start of the coaching. Further, coaches report positive effects in terms of patients’ improved knowledge and confidence, and the strengthening of the network around them. These results are in line with other studies that show improvements in various health measures and reductions in hospital admission risks of 15 to 63% [43,46–49]. However, several other studies show no improvement, and sometimes even a small increase in hospitalisations [50–53]. A possible explanation for our findings may lie in the fact that coaching in the reported programme was highly intensive, aimed at providing safety and trust, and was conducted by specialised, experienced respiratory nurses, rather than more generally trained nurses or medical assistants as often reported in other studies.

Despite these promising outcomes, agreements on how to structurally implement the improvements derived from the coaching project are yet to be made. Further, since several of the purchaser’s staff involved have left the organisation, there appears to be little initiative to evaluate the project and make long-term agreements. This is noteworthy since the successful implementation provides an opportunity for the purchaser to benefit from the investments already made. Again, this highlights the purchaser’s difficulty to sustain the orchestrator’s logic and establish coherent thinking within the organisation.

### Stakeholder pressures and the purchaser’s internal response

**Fig 1:**
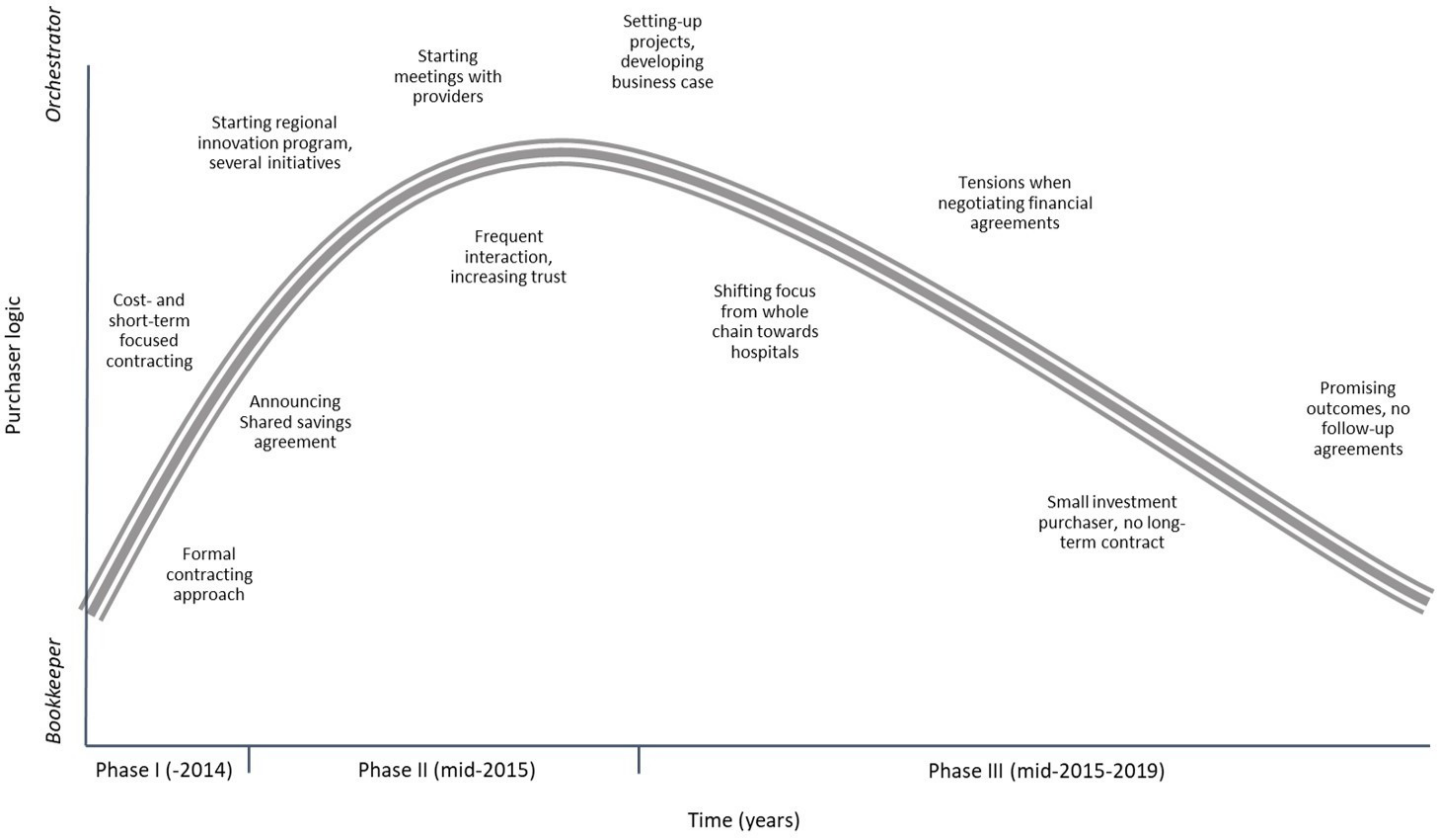
The emergence of the orchestrator’s logic and falling back into the bookkeeper’s logic.

The previous section describes three phases showing how the purchaser adopted the orchestrator’s logic but subsequently fell back into the bookkeeper’s logic (Fig 1). The table in S2 File provides a more detailed overview of the expressions of both logics and relates these to the various pressures exerted by care providers, policyholders and the government. Each of these stakeholder groups exerts institutional pressure on the purchaser: relationship pressures, cost pressures, medical demands, public health demands and uncertainty. We found that care providers especially exert relationship and uncertainty pressures. For example, the relationship between providers and the purchaser has tensions due to conflicting financial interests that drive the purchaser’s bookkeeper’s logic. Nevertheless, when the relationship improved, this encouraged an orchestrator’s logic. Pressure due to uncertainty emerged because the purchaser had little insight into the care provided and related costs. From the public, the purchaser comes under medical and cost-related pressures which are often conflicting. Ill patients expect the purchaser to provide treatment but, in general, citizens want to pay low insurance premiums and may switch to another insurer with a lower premium, thereby driving a bookkeeper’s logic. The government exerts cost, medical and public health pressures. From a societal perspective that health insurers are expected to control the increasing healthcare costs but, at the same time, guarantee accessible, high-quality care. Our study shows that the purchaser primarily focusses on the cost-containment goal, but that they are also sensitive to public health and medical demands which drove the emergence of the orchestrator’s logic.

The different stakeholder pressures explain the changes in the purchaser’s logic over time. Notably, we also observed that it is the purchaser’s intra-organisational alignment that determines how it responds to these pressures. When it came to contracting, the primary and the secondary care departments had separate responsibilities and only communicated to a limited extent about the COPD coaching project. This was why the purchaser did not make agreements with care providers along the care chain. In effect, the project was predominantly organised and financed from a secondary care perspective, and no long-term agreements were made. From a functional perspective, we saw that contracting managers only involved medical and policy advisors to a limited extent. Due to this lack of involvement of medical advisors, the purchaser’s short-term financial focus persisted, and explains their reluctance to invest in long-term improvements for COPD patients’ health. Moreover, at the beginning of this study, we saw that the closely involved policy advisors were able to facilitate the COPD coaching project and build up relationships with providers but, later, only contracting managers remained involved and these generally showed less commitment to the project, which slowed progress. This damaged the already fragile relationships with the providers and created greater uncertainty, pushing the purchaser back towards a bookkeeper’s logic.

**Fig 2:**
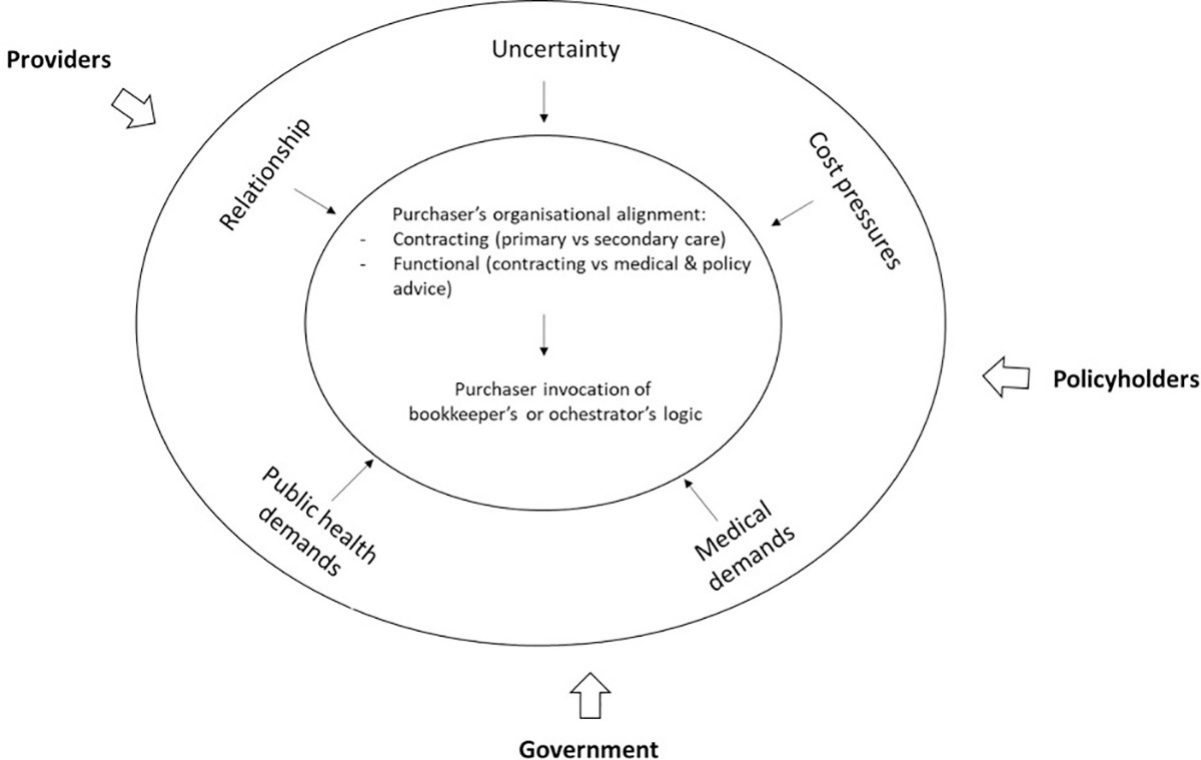
Overview of the different stakeholders, their pressures and the purchaser’s internal response.

Fig 2 summarises the different stakeholders, pressures and the purchaser’s internal responses, which are then further discussed in the next section.

## Discussion

The pattern observed, showing the expression of good intentions and supportive actions, but also the purchaser’s inconsistent and symbolic behaviour, illustrates an often-observed phenomenon in healthcare where innovative projects are frequently initiated but subsequently fail to become structurally embedded in the care chain or system [6–8]. In this study, we longitudinally mapped stakeholder pressures and their influence, thereby explaining the purchaser’s initial adoption of the orchestrator’s logic and subsequent falling back into the bookkeeper’s logic. The patterns revealed enabled us to establish two explanatory mechanisms, providing an understanding as to when, how and why the purchaser adopts these logics. First, we found that the various stakeholder pressures, which vary in strength over time, interact in influencing the purchaser. Second, we saw that the purchaser’s internal organisation influences how it deals with these stakeholder pressures. Below we advance two pairs of propositions which are in line with the phases described in the results section (see also Fig 1).

### Interactions among stakeholder pressures

In the first mechanism, the purchaser’s logics are driven by the interactions between the various stakeholder pressures. Cost pressures were clearly a driver of the purchaser’s logic. During the process of setting up the COPD coaching project, the purchaser was initially reluctant to invest in the project and demanded a strong business case. Interestingly, as the talks between the project members continued, the purchaser developed an increasingly flexible attitude. For example, although delivering a sound business case remained challenging, the purchaser’s employees involved appeared willing to defend the project within their organisation to enable its continuation. Thus, we find that when relationship pressures are eased, the purchaser became less sensitive to cost pressures and uncertainty. Conversely, we observed that persistent pressure to control care expenses reduced the sensitivity of the purchaser to medical and public health demands. The purchaser initially showed increasing awareness of the importance of improving care for COPD patients, and its employees understood that better health outcomes would not only benefit patients and society but could also reduce costs. Nevertheless, one year after the first meetings, due to uncertainties about future care costs and outcomes, the purchaser decided not to agree to a regional shared savings contract, showing that adoption of the orchestrator’s logic had halted.

Thus, the first explanatory pattern shows that the purchaser’s institutional logic is driven by the various interacting stakeholder pressures. Based on these findings, we advance the following propositions:

**Proposition 1a:**

*The emergence of the orchestrator’s logic is driven by medical and public health demands and improved purchaser*-*provider relationships that lower purchaser sensitivity to uncertainty and cost pressures*

**Proposition 1b:**

*The predominant orchestrator’s logic is attenuated by the persistent pressure to control short-term costs*, *which lowers purchaser sensitivity to medical and public health demands and increases sensitivity to uncertainty*

The observed failure to maintain the momentum in developing into a strategic purchaser shows similarities to studies on managed competition in a US context. Here, the initially fruitful development of Health Maintenance Organisations (HMOs) led to a ‘managed care backlash’: purchasers, providers and governments ending up in a cumbersome collaboration without sustained improvements for patients [54,55]. We have studied this issue in the similar Dutch context of managed competition, where independent purchasers and providers are responsible for organising and providing high-quality care within the available budgets. Our explanatory patterns provide a better understanding of the challenges in achieving sustainable care improvements through purchasing that are seen in various care contexts and countries. The external demands from multiple stakeholders trigger purchaser behaviour that follows a typical pattern of the emergence of the orchestrator’s logic, along with setting up improvement projects, followed by falling back into the bookkeeper’s logic and a failure to structurally embed care improvements. Elsewhere, healthcare management and health services scholars have argued that purchasers’ development of their role as a strategic purchaser in a context of managed competition is an ongoing, maturing process [2–4,30]. Our findings indeed show that maturing may take place, in the form of an emerging orchestrator’s logic. However, given the conflicting stakeholder pressures provided by the healthcare market, falling back into the default bookkeeper’s logic is likely. Moreover, as we highlight below, alignment within the internal purchaser organisation is an important requirement to sustain strategic purchasing.

### The purchaser’s intra-organisational alignment

The second mechanism, which highlights the importance of the purchaser’s own intra-organisational alignment, further explains the pattern of an emerging orchestrator’s logic and subsequent falling back into the bookkeeper’s logic. The first thing we observed was a fundamental division within the purchaser between contracting departments that focused either on primary or on secondary care services, with little communication between them. Second, when the purchaser needed to make internal decisions, there was little cross-functional collaboration between policy advisors and contracting managers. The contracting managers also only involved medical advisors, who are generally supportive of innovation, care improvement and preventive care, to a limited extent. As the project developed, the purchaser’s employees became less involved in the project, were reluctant to provide data for the project evaluation and did not agree on continuing with improvements made in the project. They appeared hesitant because of the costs involved and the uncertain outcomes of the project. This contrasted with the initial positive evaluation of the project, which indicated an increase in patient well-being and a reduction in the number of hospital readmissions and associated costs. Thus, this uncertainty and hesitance arose at least in part because of the purchaser’s lack of internal communication. The inconsistent intra-organisational behaviour and structure explain the falling back into the bookkeeper’s logic, but also illustrate the delicate balance between developing and sustaining the orchestrator’s logic or falling back into bookkeeping. Nevertheless, the initially created momentum and success achieved by the project highlight the opportunity to achieve a sustained adoption of the orchestrator’s logic.

This identified pattern shows that the extent of intra-organisational alignment influences how the purchaser deals with external stakeholder pressures. This leads to our second pair of propositions:

**Proposition 2a**:

*Falling back into the bookkeeper’s logic is driven by a lack of intra*-*organisational alignment that increases the purchaser’s sensitivity to uncertainty and cost pressures*

**Proposition 2b**:

*Sustained adoption of the orchestrator’s logic is more likely when the purchaser*’*s internal organisation is aligned since this increases the purchaser’s sensitivity to medical and public health demands*

The first pair of propositions attempt to explain the gradual development of strategic purchasing based on the dynamics between external stakeholder pressures, and we now connect the purchaser’s internal responses to these pressures. If purchasers want to sustainably develop an approach based on strategic purchasing, this desire needs to be widely shared and structurally developed within their organisation. Here, we build further on healthcare purchasing studies that particularly emphasise the importance of providing the right purchasing tools or market characteristics for purchasers to take on an orchestrating role [4,9,10,18]. As argued by Donato [37], these studies, often theoretically grounded in TCE, give only limited attention to the resources and capabilities developed within a firm. As our study established, such capabilities, in the form of trustworthy relationships, clinical knowledge and the tools and capacity necessary to measure outcomes, are pivotal in being able to deal with the various demands placed by the purchaser’s stakeholders. Revealing the relationships between such capabilities and stakeholder demands contributes to understanding when, how and why purchasers who have developed an orchestrator’s logic may be inclined to fall back into their traditional role of a bookkeeper.

On a positive note, one could anticipate that, provided a purchaser is able to develop its internal capabilities, a contrasting, self-enforcing spiral that supports clinical improvement and improving purchaser-provider relationships should be possible. This possibility can maybe be seen in the relatively successful implementation of Accountable Care Organisations (ACOs) in the US, where a long-term strategy was accompanied with considerable attention being given to measuring care outcomes and the continuity of relationships [56]. Further investigation into if and how our propositions apply in such contexts of managed competition could prove valuable for healthcare management.

### External and internal dynamics of institutional logics

The institutional logics perspective adopted in our study has contributed to unravelling the subtle dynamics that take place as external demands and relationships develop. Earlier research on institutional logics showed that the external environment, for example through regulation, affects the logics of organisations [13,14]. It has been illustrated how organisations incorporate different elements of, for example, commercial and professional logics in order to create internal and external legitimacy [16,57]. Our study shows a similar, yet subtle, pattern. Although public health demands encouraged the purchaser to invest in better chain-wide care delivery and health outcomes, as the project developed, the purchaser’s involvement and support became more symbolic. It seemed that demonstrating to the public and policymakers that they were able to successfully implement a project became more important than actually achieving better health outcomes.

It is generally accepted that individuals are able to adapt their thinking, which enables the gradual development of new logics [14–16]. Indeed, we saw that individuals were flexible in their daily use of logics, switching between their own beliefs and the demands of their organisation or of external stakeholders. However, in order to explain changing institutional logics, we believe that more attention should be given to structural aspects. We found that the limited collaboration between departments within the purchasing organisation increased the scepticism of several of its contracting managers regarding collaboration with providers. Consequently, the organisation became less proactive in setting up improvement initiatives. A reversed pattern may also take place: if more people within the purchaser became involved, a more trustworthy and proactive attitude towards collaborating with providers develops.

The patterns observed show that people do not only navigate between various external demands strategically, as is often argued by institutional logics scholars [13,15,16]. The purchaser’s own internal organisation also plays an important role in shaping day-to-day actions and sometimes leads to self-enforcing mechanisms since the purchaser’s response may either strengthen or attenuate some of the pressures already present. This explains the typical patterns identified within organisations struggling with conflicting short- and long-term goals, not only in healthcare but also, for example, in the context of environmental sustainability [58,59].

### Policy and managerial implications

Policymakers often expect the market to stimulate purchaser–provider collaboration and the development of agreements that align financial incentives between buyers and providers and between providers themselves [4,18,60,61]. We show that this may be overly optimistic as healthcare purchasers can be in a difficult position being embedded in care chains with stakeholders that exert a range of pressures. These pressures can lead to inconsistent or counterintuitive behaviour because pressures change over time and affect how the purchaser deals with each pressure. Policymakers should thus seek to create a stakeholder environment that stimulates the adoption of the orchestrator’s logic. This requires increased transparency of costs and care outcomes to reduce uncertainty when pursuing care chain improvement. In terms of creating the right financial incentives, a positive example may be the national financial framework agreements initiated by the Dutch government. These agreements set the available budgets for different care sectors, such as primary, secondary and social care. Here, for example, hospitals and purchasers now have a joint financial interest as the framework demands a budget shift from secondary to primary care.

The purchaser itself can also manage how it is influenced by stakeholder pressures. We saw that the purchaser and providers can sometimes end up in a negative cycle that complicates establishing collaborative relationships. We also saw a positive example of this mechanism when physicians, the purchasing organisation and the provider’s managers established a better relationship when jointly setting up the COPD coaching project. The resulting increased trust seemed to convince senior employees in the purchasing organisation that the project would be successful and that investing in it would pay-off in the future. As part of such a collaborative process, the purchaser should invest in their medical knowledge, data analysts and process support staff when trying to develop an orchestrator’s strategy. Given the importance of clinical knowledge and of good relationships with providers, one could argue that having an employee responsible for contracting overall COPD care (i.e., contracting care from the full range of providers) would support the purchaser in sustaining an orchestrator’s role. Care providers should also take note of the described mechanisms. Elsewhere in the Netherlands, there have already been promising examples where hospitals took the initiative for a more chain-wide way of delivering care [62]. Here, by setting aside the tensions over financial interests, the purchaser and the providers were able to encourage care improvements.

### Limitations

This case study provides valuable insights into the healthcare purchaser’s potential role as a care chain orchestrator. Nevertheless, the study has a number of limitations. First, we only studied one care chain where only small-scale initiatives were taking place. As such, we were limited by the specific context and possibly incidental circumstances of the case studied, which reduces the generalisability of our findings. Nevertheless, our unique case, studied with a longitudinal design, has provided a better understanding of the mechanisms that shape the purchaser’s logics over time. This level of understanding requires an in-depth study of what can be very context-specific incidents, actions and perceptions as they occur. Although we believe that the mechanisms that explain why the purchaser initially adopted a new institutional logic and then fell back into the old, default logic, are at least partly generalisable to other contexts, this needs to be confirmed in future studies, which may for example pay attention to the aspect of national culture [63].

Second, a downside of our decision to limit our study to the COPD care chain, so as to clearly demarcate our case study, is that, in COPD care, there is an ongoing debate on how best to treat patients [64]. For other care types, such as diabetes, where procedures are more straightforward and where suggestions for improvements create less discussion among stakeholders, the collaborative process may be easier. However, the healthcare field inevitably has to deal with clinical uncertainty since each disease type, region and organisational setting has its own unique properties. Further, COPD care has several options for improving health outcomes and reducing costs and so, if anywhere, one could expect healthcare purchasers to take a proactive, strategic role here. The COPD case study therefore provided a good research context for us to clearly identify the organisational mechanisms that result from such a challenging situation.

### Conclusion

We have shown how the pressures exerted by various stakeholders and the purchaser’s own internal organisation shape the purchaser’s adoption of institutional logics. Improving purchaser–provider relationships facilitates the adoption of an orchestrator’s logic, while pressure to control costs limits this process and explains the falling back into a bookkeeper’s logic. Our research provides an improved understanding of the purchaser’s difficult position in the care chain that hinders it in adopting an orchestrator’s role. Nevertheless, we do see the potential of adopting an orchestrator’s logic and thereby the purchaser taking a proactive role as the funder of chronic care services. Building knowledge, better purchaser–provider relationships and reducing uncertainty through information exchange and collaboration can create a positive cycle that allows the orchestrator’s logic and chain-wide care improvement itself to sustain. Policymakers can support such a development by supporting transparency of care outcomes and costs and by reducing conflicting financial interests between purchasers and providers so that the healthcare system can function as intended.

## Supporting information

S1 File Interview guide. (DOCX)

S2 File Illustrative quotes and coding scheme. (DOCX)
