# Supplementary material for: Orchestration versus bookkeeping: How stakeholder pressures drive a healthcare purchaser’s institutional logics
Source: PLoS One. 2021 Oct 13;16(10):e0258337. doi: 10.1371/journal.pone.0258337 (PMC8513887; doi:10.1371/journal.pone.0258337)
Supplement: S1 File — (DOCX) [file pone.0258337.s001.docx]

**Supporting file 1. Interview guide.**

**Orchestration versus bookkeeping: how stakeholder pressures drive a healthcare purchaser’s institutional logics**

The interviews were primarily aimed at the collaborative process on a COPD out of hospital coaching initiative between regional care providers and the largest regional health insurer. Interviewees were directly involved in the project, or had a more general role (managerial, medical professional) in regional care delivery. Besides focusing on the specific project, interviewees were asked to reflect on general health system, policy and practical issues related to improving chain-wide care delivery. The interview guide consists of seven main questions. Based on the responses, the interviewees were asked follow-up questions on their opinion and perceptions about topics shown in italic/gray.

*At the start of the interview, the interviewer explains the goal and subject of the interview, asks the participant to provide written consent for participation in the study and recording of the interview.*

1. How do you look back at the process of the COPD coaching project as it has developed so far?
2. Which moments did you perceive as vital during the project team meetings and why?

- Discussion about need for a project manager and required time of pulmonologists.
- Role of physiotherapy in the project.
- Setting up of a business case.
- Not involving general practitioners (why was this, what are barriers regarding primary and secondary care collaboration?).
- Lack of clarity about financial agreements.

1. What were your expectations about this project on forehand, and did the project so far deliver what you expected?

- The lack of agreement on a shared savings contract. How do you explain this?
- What do you think about the current financial agreement for this project?

1. Do you think this project will meet the expectations of each of the involved parties?
2. How do you perceive the collaborative process within the project team?

- Does everyone stick to made agreements? Why, why not?
- Are there any barriers for collaboration?
- In case (a lack of) trust is being mentioned. How do you explain this? Has trust changed over time?

1. To what extent has this project been discussed within your organisation? Why, why not?

- Communication between pulmonologists and hospital management.
- Communication between purchaser policy advisors and management / contracting department.
- Is this project supported within the organisation, why, why not?

1. Traditionally, health insurers negotiated only with hospital management, now direct meetings between medical specialist partnerships and insurers take place. What are the implications of this?

How does the hospital management deal with this?

How does this affect the relationships between involved parties (insurer, medical specialists, hospital management)?
