# Supplementary material for: Orchestration versus bookkeeping: How stakeholder pressures drive a healthcare purchaser’s institutional logics
Source: PLoS One. 2021 Oct 13;16(10):e0258337. doi: 10.1371/journal.pone.0258337 (PMC8513887; doi:10.1371/journal.pone.0258337)
Supplement: S2 File — (DOCX) [file pone.0258337.s002.docx]

**Supporting file 2 of paper: Orchestration versus bookkeeping: how stakeholder pressures drive a healthcare purchaser’s institutional logics**

**Illustrative quotes and coding scheme.**

*Overview of purchaser actions over time, how they link to the orchestrator’s and bookkeeper’s logics and how they can be explained by the different stakeholder pressures: (R) Relationship pressures, (C) Cost pressures, (M) Medical pressures, (P) Public health demands, (U) Uncertainty*

| **Action** | **Institutional logic** | **Stakeholder pressures** | | | **Representative quote** | **Outcome** |
| --- | --- | --- | --- | --- | --- | --- |
|  |  | **Government** | **Providers** | **Policyholders** |  |  |
| Cost and short-term focused contracting | Bookkeeper |  | Little mutual trust (R) | Demanding low insurance premiums (C) | *“The negotiations take place at a highly aggregated level. (Insurers say): this year you received xx amount of money, so next year we will reduce or increase that amount a bit”* Hospital department manager 1 | Little room for improvement initiatives |
| Formal  contracting approach | Bookkeeper | Regulation aimed at administrative role (C, M) |  |  | *“Here are people who don’t know any more than, ‘I need to contract a care provider, so I need to compile a list of requirements.’ Well, after two years you understand how that works, namely, it is the same every year”* Purchaser policy advisor | Innovative projects are rare |
| Announcing intention to use shared savings agreements | Orchestrator | Government support for long-term improvement (P) |  | Pressure to improve chronic care delivery (M) | *“As part of these contracts, we look at substitution, which care services can go where?... We are on the eve of pushing changes. It is no longer a question of if we want to do that. It needs to be done…As part of our Vanguard status, contract innovation is possible, for example we work with shared savings agreements in mental care and surgical care”* Interview reported in news media with CEO of Health Insurer 2015 | Providers motivated to initiate improvement initiatives |
|  |  |  |  |  |  |  |
| Starting regional innovation program – several improvement initiatives | Orchestrator | Government support for long-term improvement (P) |  | Pressure to improve chronic care delivery (M) | *“We are mainly interested in the quality of care and the premise that lies behind it. You often see that when you focus on the right care at the right place, it is by definition cost-saving”* Purchaser Policy Advisor 2 | Providers motivated to initiate improvement initiatives |
| Initiating  meetings with providers | Orchestrator | Government support for long-term improvement (P) |  | Pressure to improve chronic care delivery (M) | *“I found it, I experienced it as very pleasant, very direct, very practical. So, no cumbersome (discussions)…no just, ‘let’s set a date, order some sandwiches and sit down together’. And that practical attitude is just what you need”* Purchaser Policy Advisor 2 | Several initiatives to improve COPD care |
| Setting-up projects, developing business case | Orchestrator | Government support for long-term improvement (P) | Well-organised pulmonologists, already some history of collaboration (R) | Pressure to improve chronic care delivery (M) | *“Yes, and at a certain point (insurer policy advisor 2) strongly supported the project and that is when trust developed”* Hospital board member 1 | COPD home coaching project plus new care pathway |
| Frequent interaction, collaboration, increasing trust | Orchestrator |  | Increasing trust in pulmonologists’ intentions (R) |  | *“I found it nice to see that there are medical specialists who dare to look beyond their own interests. As they said, they didn’t have to do it, they will get their salary anyway, putting it very bluntly (…) So that I really appreciated it, for me personally that means that I put a bit of extra effort into arranging things”* Purchaser Policy Advisor 2 | Constructive relationships |
| Shifting focus  from the whole chain towards hospitals | Bookkeeper |  | Poor relationships with GPs (R) | Need for short-term results (M) | *“I think we are late with involving the primary care providers. (…) Because that is often a bit of a fear, that they feel side-lined (…) But, on the other hand, if we explain it well, then it doesn’t have to be a problem. However, the more people that are involved in the design phase (of the project), the more complex it will become”* Pulmonologist 1 | Excluding GPs |
| Small investment by purchaser on coaching project, no long-term/shared savings contract | Bookkeeper | Need for short-term results (P) | Lacking insight into providers’ care costs and volumes (U) | Need for short-term results (M) | *(Discussing shared savings) While this is about public money that, you know, is saved and then you’re going to give that money to entrepreneurs (the pulmonologists) who actually have a partnership. So you’re giving them public money as a sort of profit while they actually (for example) prescribe less medicine.* Purchaser Policy Advisor 2  *“Because it could be good for the patient, that they receive coaching, but, in the end, it is hard to determine if the business case is positive”* Purchaser Policy Advisor 2 | COPD coaching project starts |
| No follow-up agreements despite promising outcomes | Bookkeeper |  | Hospitals hesitant due to increased work pressure, lack of financial incentive (C) | Pressure on care budgets, insurance fees (C) | *(discussing the follow-up of the project) No, we have not addressed that yet. That is actually not urgent if you ask me. So, it depends on the outcomes of the evaluation how you organise it in the future”* Purchaser financial controller | Increased satisfaction of patients and care professionals  COPD coaching project on-hold |
